# Acceptability of immersive 360-simulation for neonatal healthcare professional education: a pilot study

**DOI:** 10.1136/bmjpo-2025-004458

**Published:** 2026-08-06

**Authors:** Jennifer L H Peterson, Natalie Gallagher, Sunaya Hirani, Ruth Gottstein, Ranganath Ranganna

**Affiliations:** 1 Faculty of Biology Medicine and Health, The University of Manchester, Manchester, UK; 2 Neonatal Intensive Care Unit, Manchester University NHS Foundation Trust, Manchester, UK; 3 Neonatal Intensive Care Unit, Liverpool Women’s Hospital NHS Foundation Trust, Liverpool, UK

**Keywords:** Neonatology, Technology, Qualitative research, Intensive Care Units, Neonatal

## Introduction

 Human factors skills are key to effective neonatal resuscitation. Perinatal professionals require training opportunities which are easily accessible, cost-effective and practical to develop and hone these skills.^1 2^ Simulation is an embedded training modality in many units. However, face-to-face simulation requires facilitator expertise, participant availability and access to simulation equipment. This can be logistically challenging.

**Figure 1:**
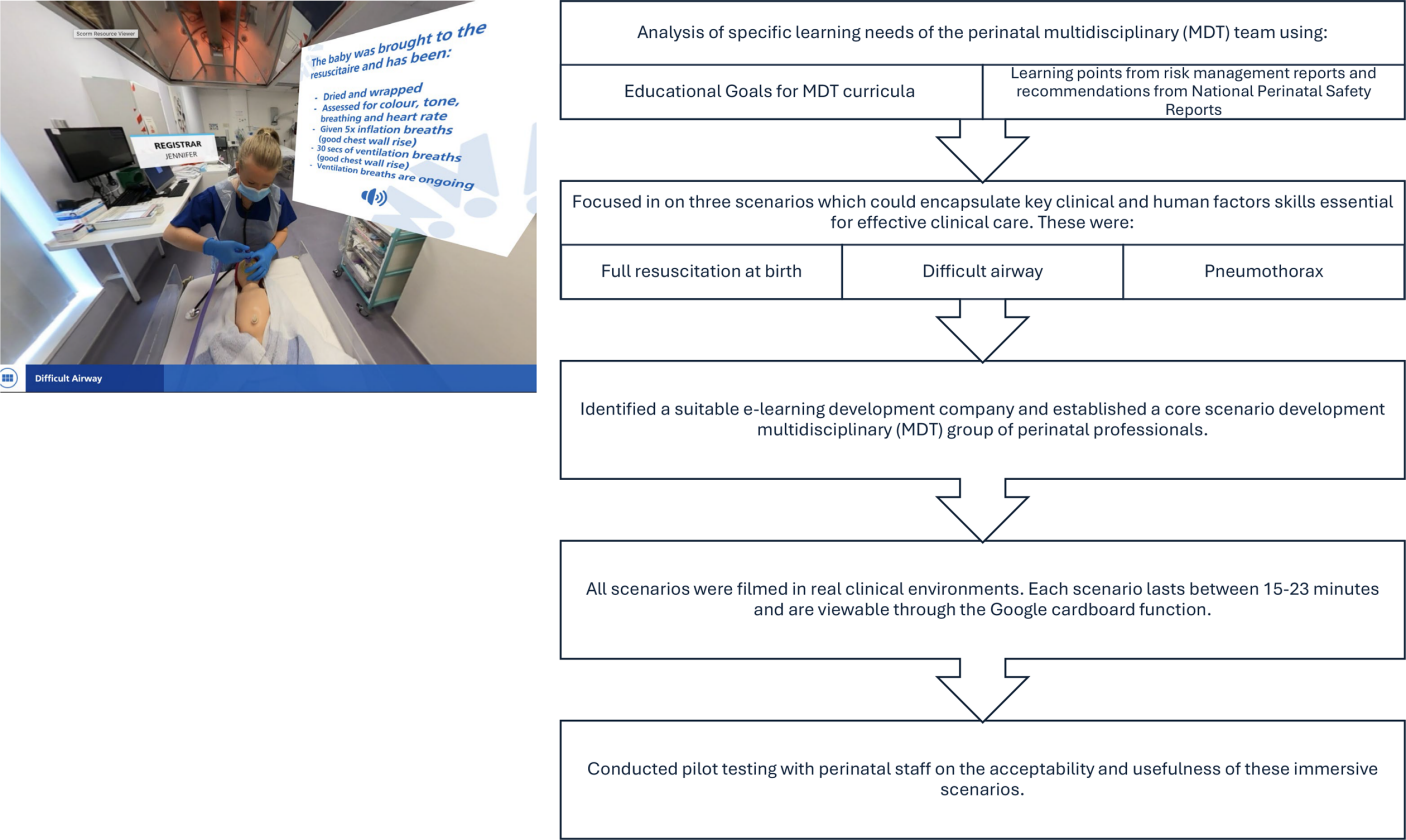
Development of the augmented 360-video simulation programme. Development was funded through a Health Education England (HEE) grant and the scenarios are hosted on the HEE Learning Hub: https://learninghub.nhs.uk/catalogue/neonatal-virtual-reality-simulation-scenarios/browse#catalogue-details

Interactive virtual reality simulation programmes are commercially available and may be effective for learners but are often costly and computer-generated, reducing the realism of the scenarios.^3 4^ Immersive 360-simulation has been shown to be a potential solution to this, allowing bespoke development of training scenarios covering a range of neonatal emergencies by local facilitators. Our simulation team developed a series of immersive, augmented 360° simulation scenarios filmed on a GoPro 360 in clinical environments using local equipment (figure 1). Learning prompts and questions, as well as additional narrated information boxes, were added on top of the scenario footage to create an immersive, engaging resource. These scenarios focused on key clinical and human factors skills, were compatible with inexpensive virtual reality headsets and could be used either individually or in facilitated groups.

The 360° simulation scenarios were a new educational modality for both facilitators and participants at our centre and therefore, an evaluation of neonatal healthcare professionals’experiences was performed.

## Methods

Between January and July 2024, the simulation education team offered professionals the opportunity to participate in a 360-simulation, inviting them to try the 360-scenarios within the weekly unit-wide simulation programme and during a 2-day simulation programme for trainee advanced neonatal nurse practitioners (ANNPs). Participation in the study was voluntary. Professionals participated in a 360-simulation and completed a post-360 evaluation form with preset questions related to their experience and rating of these scenarios for clinical and human factors training. Professionals were recruited from across the neonatal team, including doctors, advanced nurse practitioners and nurses. All staff recruited to the study had participated in face-to-face simulation at least once in the 12 months prior to the 360-session.

## Results

The study recruited 51 professionals with a mixture of neonatal doctors (n=13), ANNPs (n=24) and nurses (n=14). All professionals reported the 360-simulations had been a useful training opportunity. There was variation in preference for using the scenarios individually (37%), in a facilitated group (20%) or in combination (35%).

**Figure 2:**
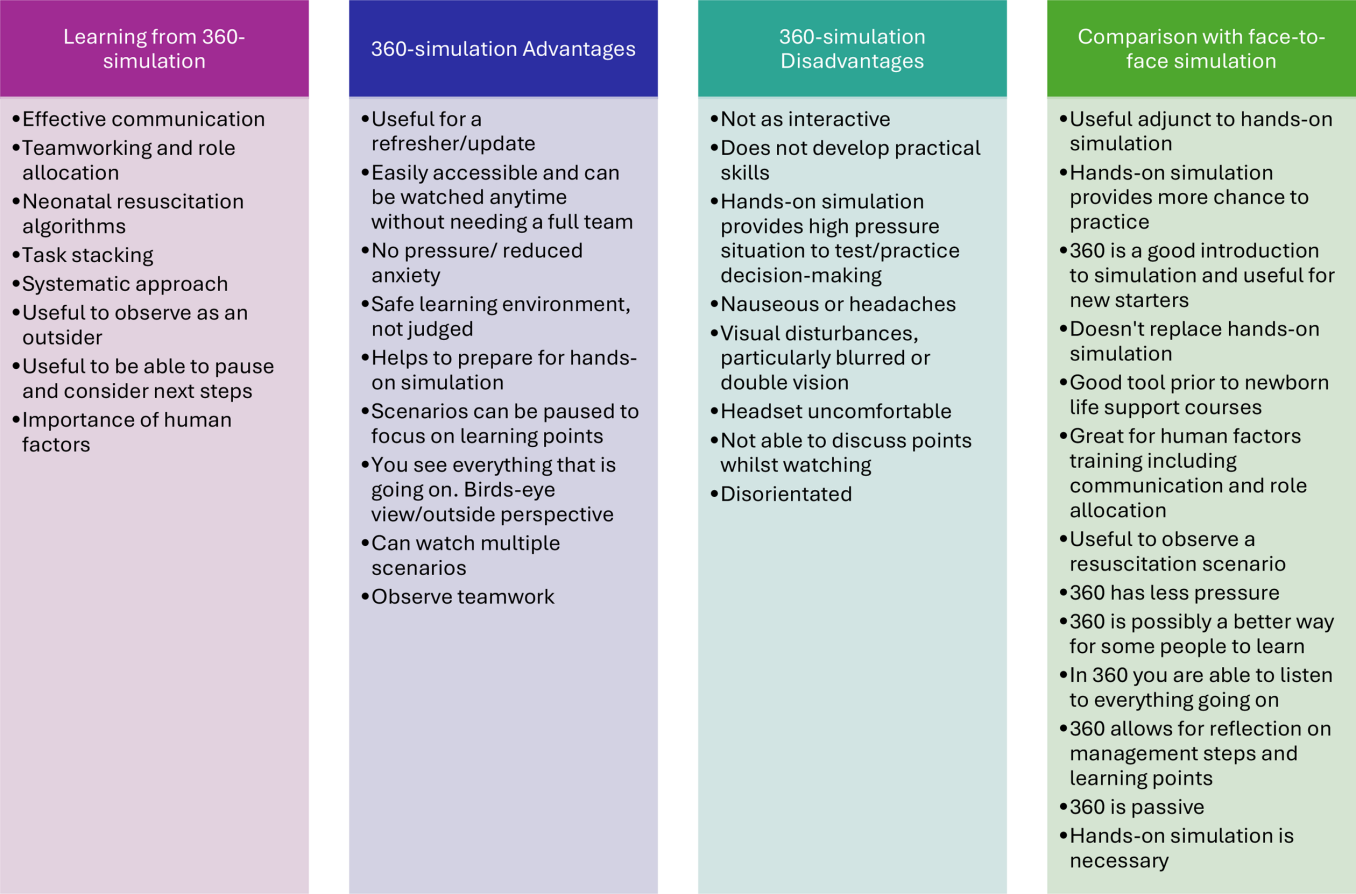
Participant qualitative feedback.

Professionals highlighted that 360-simulation allows the participant to watch the scenario multiple times focusing on different team member roles and communication styles (figure 2). Participants appreciated the opportunity to adopt an ‘outsider’ perspective within the scenarios as this enabled identification of important human factors elements, such as role allocation and task stacking behaviours, aiding participant understanding of effective team dynamics and organisation. Additionally, feedback indicated that the 360-simulations were perceived as less intimidating than face-to-face sessions and that it was useful to be able to pause the scenarios and consider the next steps for management. Feedback showed that participants felt the 360-simulations were a safe learning space and it was advantageous that they were easily accessible and, once launched, required fewer resources to set-up the training session than gathering all the equipment and personnel for a face-to-face simulation.

Reported disadvantages of 360-simulation included initial disorientation when entering the 360° environment and the lack of procedural skills development which would likely be better suited to face-to-face sessions (figure 2). Additionally, there were concerns about potential nausea when using the 360-scenarios in headset mode; however, this complication was not encountered during the sessions.

## Discussion

This evaluation suggests that 360-simulation is both an acceptable educational approach for neonatal staff and a feasible adjunct to face-to-face training, with each simulation modality contributing to different but complementary components of learning.
