# Supplementary material for: Acceptability of immersive 360-simulation for neonatal healthcare professional education: a pilot study
Source: BMJ Paediatr Open. 2026 Aug 6;10(1):e004458. doi: 10.1136/bmjpo-2025-004458 (PMC13448597; doi:10.1136/bmjpo-2025-004458)
Supplement: Supplementary file 1 [file bmjpo-2025-004458.reviewer_comments.pdf]

PEER REVIEW HISTORY

BMJ Paediatrics Open publishes all reviews undertaken for accepted manuscripts. Reviewers are asked to complete a checklist review form and are provided with free text boxes to elaborate on their assessment. These free text comments are reproduced below.

ARTICLE DETAILS

**TITLE (PROVISIONAL)**	Acceptability of immersive 360-simulation for neonatal healthcare professionals education: a pilot study
**AUTHORS**	Peterson, Jennifer<br>Gallagher, Natalie<br>Hirani, Sunaya<br>Gottstein, Ruth<br>Ranganna, Ranganath

VERSION 1 – REVIEW

**REVIEWER NAME**	Arnold, Jennifer
**REVIEWER AFFILIATION**	Boston Children's Hospital, Pediatrics
**REVIEWER CONFLICT OF INTEREST**	No Conflict of Interest
**AI DISCLOSURE**	No, I have not used AI
**DATE REVIEW RETURNED**	25-Jan-2026

**GENERAL COMMENTS**	This paper is a relevant and important topic. It is well written. The changes i would recommend would be related to the methodology. It is difficult to understand what the VR experience actually is based on the conflict of VR with what i think is being described as 360 video experiential learning. It is a misnomer to call the training a VR training as VR refers to an interactive computer generated simulation experience where the learner/participant can make decisions and see consequences in the virtual enviornment. What appears to be described here is a 360 video experience where the learning is passive. I would recommend the authors be more explicit in the description of the learning modality itself, how it works, and what it's limitations and functionalities are. It is misleading to refer to it as virtual reality. I would also recommend that they propose how to incorporate this type of learning into a broader neonatal resuscitation curriculum. Lastly, i would recommend some description of how other organizations might replicate creating this type of 360 video learning experience to show its broader applicability.

VERSION 1 – AUTHOR RESPONSE

Reviewer comments:
Comment: This paper is a relevant and important topic. It is well written.
Response: Thank-you.

Comments: The changes I would recommend would be related to the methodology. It is difficult to understand what the VR experience actually is based on the conflict of VR with what i think is being described as 360 video experiential learning. It is a misnomer to call the training a VR training as VR

refers to an interactive computer generated simulation experience where the learner/participant can make decisions and see consequences in the virtual environment. What appears to be described here is a 360 video experience where the learning is passive. I would recommend the authors be more explicit in the description of the learning modality itself, how it works, and what its limitations and functionalities are. It is misleading to refer to it as virtual reality.
Response: We have updated throughout the article to reflect that we are referring to immersive, augmented 360-video simulations. Augmentation was with pop-up questions, learning points and additional information. We have included a screenshot from one of the scenarios in Figure 1 to demonstrate this. The image utilises one of the author's faces (JP) who has given consent for the image to be used in this publication.

Comment: I would also recommend that they propose how to incorporate this type of learning into a broader neonatal resuscitation curriculum.
Response: Given the word count this was felt to be beyond the scope of this article which was to provide educators with evidence that this type of training is useful to neonatal professionals.

Comment: Lastly, I would recommend some description of how other organizations might replicate creating this type of 360 video learning experience to show its broader applicability.
Response: We have more clearly outlined the development and funding of these 360-simulation scenarios. We have included details in the Methods section and Figure 1 about how we approached developing these scenarios to allow other educators to model a similar process if they choose.

VERSION 2 – REVIEW

**REVIEWER NAME**	Rockwell, Nick
**REVIEWER AFFILIATION**	Children's of Alabama
**REVIEWER CONFLICT OF INTEREST**	No Conflict of Interest
**AI DISCLOSURE**	No, I have not used AI
**DATE REVIEW RETURNED**	09-Jun-2026

**GENERAL COMMENTS**	The statement in discussion: "360-simulation is acceptable to neonatal staff as an education modality and can be a useful adjunct to face-to-face sessions with each simulation modality targeting different, complementary aspects of training"<br>-You don't have information to determine if it is a useful adjunct to face-to-face sessions. Your data would agree with it being an acceptable modality but the data doesn't show if it was useful or not. No skills were assessed before and after. And some feedback still suggested hands on is necessary. You showed that this modality is feasible.<br><br>Other adjustments to responses seems appropriate.

VERSION 2 – AUTHOR RESPONSE

Response to reviewer:

Reviewer comment:

The statement in discussion: "360-simulation is acceptable to neonatal staff as an education modality and can be a useful adjunct to face-to-face sessions with each simulation modality targeting different, complementary aspects of training"

You don't have information to determine if it is a useful adjunct to face-to-face sessions. Your data would agree with it being an acceptable modality but the data doesn't show if it was useful or not. No skills were assessed before and after. And some feedback still suggested hands on is necessary. You showed that this modality is feasible.
Other adjustments to responses seems appropriate.

Author response:

Thank you for your review and comment.

We agree with the reviewer and have amended the final statement to not include the term 'useful'.

This statement has been changed to 'This evaluation suggests that 360-simulation is both an acceptable educational approach for neonatal staff and a feasible adjunct to face-to-face training, with each simulation modality contributing to different but complementary components of learning.', which we hope you will find a more appropriate phrasing.

VERSION 3 – REVIEW

**REVIEWER NAME**	**Rockwell, Nick**
**REVIEWER AFFILIATION**	Children's of Alabama
**REVIEWER CONFLICT OF INTEREST**	No Conflict of Interest
**AI DISCLOSURE**	No, I have not used AI
**DATE REVIEW RETURNED**	09-Jul-2026

**GENERAL COMMENTS**	Thank you for the adjustment, this satisfies the comment.
